# Evaluating the impact of mandatory indications on antibiotic utilization in a community hospital

**DOI:** 10.1017/ash.2022.260

**Published:** 2022-07-15

**Authors:** April J. Chan, Rosane Nisenbaum, Mark Downing, Bradley J. Langford

**Affiliations:** 1 Unity Health Toronto, Ontario, Canada; 2 University of Toronto, Ontario, Canada; 3 Li Ka Shing Knowledge Institute, Ontario, Canada; 4 Applied Health Research Centre and MAP Centre for Urban Solutions, Ontario, Canada; 5 Public Health Ontario, Ontario, Canada; 6 Hotel Dieu Shaver Health and Rehabilitation Centre, Ontario, Canada

## Abstract

**Objective:**

We evaluated the impact of introducing a mandatory indication field into electronic order entry for targeted antibiotics in adult inpatients.

**Design:**

Retrospective, before-and-after trial.

**Setting:**

A 400-bed community hospital.

**Interventions:**

All adult electronic intravenous (IV) and enteral orders for targeted antibiotics (moxifloxacin, ciprofloxacin, clindamycin, vancomycin, and metronidazole) had a mandatory indication field added. Control antibiotics (amoxicillin-clavulanate, ceftriaxone and piperacillin-tazobactam) were chosen to track shifts in antibiotic prescribing due to the introduction of mandatory indication field.

**Methods:**

Descriptive statistics were used to summarize the primary outcome, measured in Defined Daily Doses (DDD) per 1000 patient days (PD). Interrupted time-series (ITS) analysis was performed to compare levels and trends in antibiotic usage of targeted and control antibiotics during 24 months before and after the intervention. Additionally, a descriptive analysis of mandatory indication fields for targeted antibiotics in the postintervention period was conducted.

**Results:**

In total, 4,572 study antibiotic orders were evaluated after the intervention. Preset mandatory indications were selected for 30%–55% of orders. There was decreased usage of targeted antibiotics (mean, 92.02 vs 72.07 DDD/1000-PD) with increased usage of control antibiotics (mean, 102.73 vs 119.91 DDD/1000-PD). ITS analysis showed no statistically significant difference in overall antibiotic usage before and after the intervention for all targeted antibiotics.

**Conclusion:**

This study showed moderate use of preset mandatory indications, suggesting that the preset list of indications can be optimized. There was no impact on overall antibiotic usage with the use of mandatory indications. More prospective research is needed to study the utility of this intervention in different contexts.

Recent evidence suggests that 30%–40% of prescribed antibiotics in hospital are inappropriate.^1^ Unnecessary usage of antibiotics can lead to selection for antimicrobial resistant pathogens, *Clostridioides difficile* infection, and other adverse events such as nephrotoxity.^2^ Documentation of indications for antibiotics is recommended to facilitate antimicrobial stewardship interventions such as prospective audit and feedback.^3^ Some studies have shown to reduce rates of inappropriate prescribing and improve medication safety^4–7^; however, more studies are needed to confirm the impact on overall antibiotic usage and shifts in antibiotic prescribing.

In October 2015, St. Joseph’s Health Centre introduced mandatory indication fields into electronic order entry for selected antibiotics. These fields acted as a force function, requiring prescribers to provide a reason for prescribing at the time of order entry before the order can be processed. We evaluated the impact of introducing this intervention on antibiotic utilization and characterized the use of predefined indications by prescribers.

## Methods

The study was conducted at a 400-bed community hospital in Toronto, Canada. An antimicrobial stewardship program (ASP) was established at our institution in 2011, with a multimodal approach including prospective audit and feedback, development of guidelines and order sets, microbiology laboratory report optimization and education. Staffing consisted of a lead ASP physician and 2 full-time equivalent ASP-trained pharmacists during the study period of October 1, 2013, to October 31, 2017.

All adult electronic intravenous (IV) and enteral orders for targeted antibiotics (moxifloxacin, ciprofloxacin, clindamycin, vancomycin, and metronidazole) had a mandatory indication field added on October 22, 2015. The first three antibiotics (ciprofloxacin, moxifloxacin, clindamycin) were selected based of risk for causing *Clostridioides difficile* infection and being not first line agents for most infections in our setting so there is higher potential for inappropriate use. The latter two antibiotics (metronidazole and vancomycin) were selected as they had more clear-cut indications. Each mandatory indication field had predefined indications tailored to the selected antibiotics as determined by the ASP team and a free-text field (Table 1). Only a single mandatory indication or free text can be selected at the time of order entry. Discrete preset indications of each antibiotic were discussed and chosen by the antimicrobial stewardship team. A descriptive analysis of the mandatory indication fields for the study antibiotics was conducted.

**Table 1.** Usage of Predefined and Free-Text Indications for Selected Antibiotics

Variable	Administration Method	
**CIPROFLOXACIN**	**PO**	**IV** ^a^
**Predefined indications, no. of orders**	219	155
Deep-seated or bacteremic gram-negative infection	36	18
Gram-negative infection and allergy to narrower-spectrum antibiotics	51	38
Gram-Negative infection resistant to narrower-spectrum antibiotics	77	59
Suspected or documented *Pseudomonas* infection	55	40
**Free-text field, no. of orders**	415	161
Specified infection	245	124
UTI/pyelonephritis	128	9
Intra-abdominal/GI	53	70
**CLINDAMYCIN**	**PO**	**IV** ^a^
**Predefined indications, no. of orders**	15	121
Adjunctive treatment of group A *Streptococcus* infection	1	22
Obligate (gut) anaerobe (consider metronidazole)	1	6
Oral anaerobe infection (not advised, consider B-lactam)	3	7
Surgical infection prevention, severe B-lactam allergy	10	86
**Free-text field, no. of orders**	36	179
Specified infection	20	92
SSTI/OM	9	…
GU	…	40
**METRONIDAZOLE**	**PO**	**IV** ^a^
**Predefined indications (no. of orders)**	257	416
Anaerobic infection	160	384
CDI	94	28
CNS infection	1	4
Parasitic infection	2	0
**Free text field (no. of orders)**	268	479
Specified infection	133	419
Intra-abdominal	68	278
GI	…	51
**MOXIFLOXACIN**	**PO**	**IV** ^a^
**Predefined indications (no. of orders)**	147	63
Respiratory infection and severe B-lactam allergy	69	26
Respiratory infection and recent use of B-lactam	60	25
Other infection (eg, skin) and severe B-lactam allergy	18	12
**Free text field (no. of orders)**	208	118
Specified infection	159	98
Respiratory	138	92
**VANCOMYCIN**	**PO**	**Enteral**
**Predefined indications (number of orders)**	171	
Recurrent CDI	102	
Severe CDI	58	
First episode of mild-to-moderate CDI (not advised)	11	
**Free text field, no. of orders**	142	
Specified infection (eg, CDI, CDI taper)	89	
**VANCOMYCIN**		**IV**
**Predefined indications, no. of orders**		452
B-lactam resistant gram-positive infection		309
B-lactam sensitive gram-positive infection, severe B-lactam allergy		82
Surgical infection prevention, severe B-lactam allergy		61
Single positive blood culture of gram-positive organism when other recent cultures are negative (not recommended)		0
**Free-text field, no. of orders**		550
Specified infection		378
SSTI/bone		87
Sepsis		73
Bacteremia		67
CNS		43
Culture results/pathogen (eg, GPC in blood, MRSA, *Enterococcus*, CNST)		119

Note. CDI, *Clostridioides difficile* infection; CNS, central nervous system; CNST, coagulase-negative *Staphylococci*; GI, gastrointestinal; GPC, gram-positive cocci; GU, genitourinary; IV, intravenous; MRSA, methicillin-resistant *Staphylococcus aureus*; NPO, nothing by mouth; OM, osteomyelitis; SSTI, skin and soft-tissue infection.

a Additional criterion of NPO in preset list of indications.

Control antibiotics (amoxicillin-clavulanate, ceftriaxone, and piperacillin-tazobactam) were chosen to track shifts in antibiotic prescribing due to the introduction of mandatory indication field because there were no specific initiatives addressing these control antibiotics during the study period.

The preintervention period was defined as October 1, 2013, to October 31, 2015, and the postintervention period was from November 1, 2015, to October 31, 2017. October 2015 was assigned to the preintervention period because the intervention started 1 week before the end of October and the assumption was that effect on antibiotic usage would be small after only a week of intervention.

Data on antibiotics were collected monthly as Defined Daily Doses (DDD) per 1000 patient days (PD). DDD was the available metric at the time and provided a longer history of antibiotic usage prior to the intervention. Descriptive statistics were used to summarize the antibiotic data. Interrupted time-series (ITS) analysis was performed to compare changes in level and slope with regard to the primary outcome. We used the regression model proposed by Wagner et al.^8^ We used Stata version 15 software (StataCorp, College Station, TX) for these analyses. ITS analysis was used to estimate regression parameters by ordinary least-squares regression-based models which accommodated ITS data. These models estimate ordinary least-squares regression coefficients with Newey-West standard errors, which handle autocorrelation and heteroscedasticity. The command “actest” was used to perform the Cumby-Huizinga tests for autocorrelation and the specific lag order up to 12.

This study was approved by the research ethics board at St. Joseph’s Health Centre on January 20, 2017.

## Results

In total, 8,399 orders were evaluated in the 1-year postintervention period, of which 4,572 orders were for targeted antibiotics and 3,287 were for control antibiotics. The preset mandatory indications were selected 30%–55% of the time, depending on targeted antibiotic (Table 1). When the free-form field was selected, the most common indication noted was a specific infection (eg, urinary tract infection-pyelonephritis for ciprofloxacin oral and intra-abdominal-gastrointestinal for ciprofloxacin IV) with very few indications that were incomprehensible (0–4 instances for each study antibiotic with examples such as a dot and a comma).

After mandatory indication field was introduced, there was decreased usage of targeted antibiotics (mean, 92.02 vs 72.07 DDD/1000-PD), driven by decreased usage of metronidazole (mean, 24.76 vs 18.44 DDD/1000-PD), ciprofloxacin (mean, 27.68 vs 21.30 DDD/1000-PD) and moxifloxacin (mean, 17.70 vs 11.89 DDD/1000-PD). We noted increased usage of control antibiotics (mean, 102.73 vs 119.91 DDD/1000-PD) driven by increased usage of amoxicillin-clavulanate (mean, 37.31 vs 43.14 DDD/1000-PD) and ceftriaxone (mean, 37.50 vs 46.88 DDD/1000-PD).

**Fig. 1.**
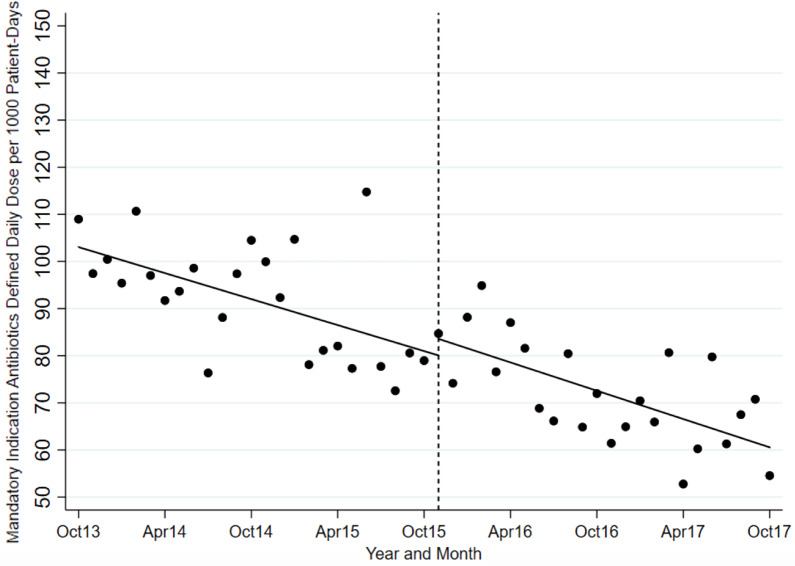
Mandatory indication antibiotic utilization pre and postintervention.

**Fig. 2.**
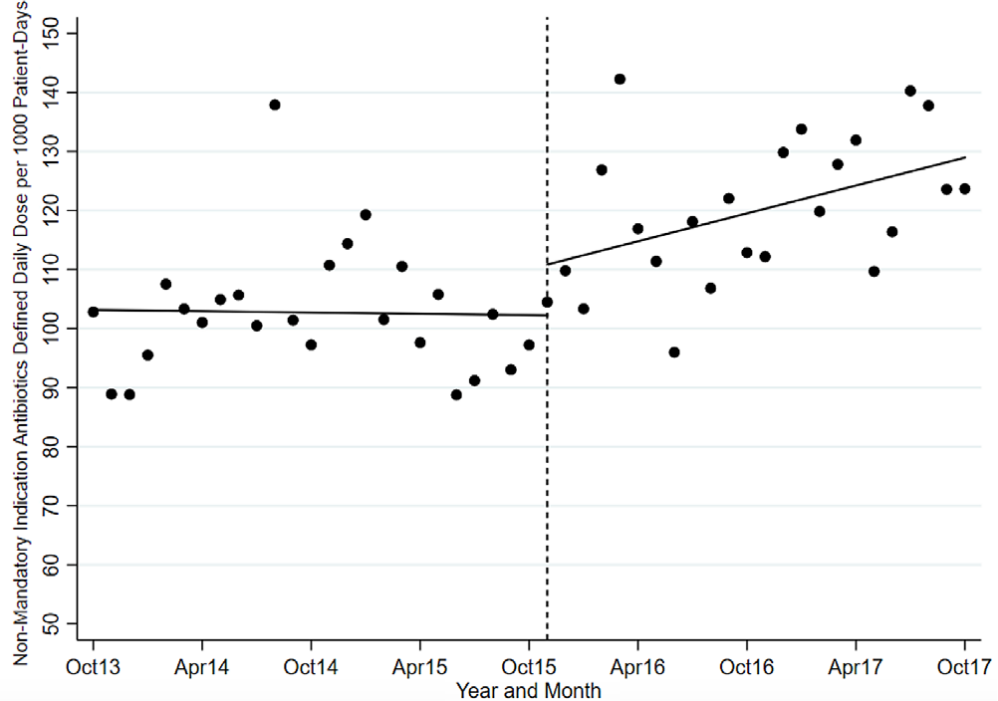
Control antibiotic utilization pre and postintervention.

The ITS analysis showed levels were not different before and after the intervention for targeted antibiotics with mandatory indications. The difference in antibiotic usage (DDD/1000-PD) before the intervention (83.58; 95% CI, 77.21–89.95) and after the intervention (80.07; 95% CI, 70.62–89.51) was 3.51 (95% CI, −7.88 to 14.90; *P* = .538). Similarly, the preintervention slope (−0.92 per month) was not different from the postintervention slope (−1.00 per month) (change, −0.08; *P* = .821) (Table 2 and Fig. 1). Considering ITS analysis for individual targeted antibiotics, moxifloxacin levels were significantly different before and after the intervention. The difference between the postintervention level (16.98; 95% CI, 14.25–19.72) and the preintervention level (11.96; 95% CI, 9.00–14.93) was 5.02 (95% CI, 1.10–8.94; *P* = .013). There was no significant change in levels or slopes for other targeted antibiotics. Similarly, ITS analysis for control antibiotics showed that levels were not different before and after the intervention. The difference between postintervention levels (110.85; 95% CI, 101.03–120.66) and preintervention levels (102.25; 95% CI, 94.74–109.76) was 8.60 (95% CI, −3.76 to 20.95; *P* = .168). However, the preintervention slope (−0.04 per month) was different from postintervention slope (0.79 per month; change, 0.82; *P* = .051). Nonetheless, given the small change in DDD/1000-PD, this is likely not clinically significant (Table 3 and Fig. 2). This trend toward increased control antibiotic prescribing was driven by a significant level change seen with piperacillin-tazobactam. The difference between the postintervention level (30.03; 95% CI, 26.67–33.38) and the preintervention level (24.41; 95% CI, 21.61–27.21) was 5.62 (95% CI, 1.25–9.99; *P* = .013).

**Table 2.** ITS Analysis (Rate of Change)

Antibiotic	Rate of Change (Pre-Intervention) DDD/1000-PD/month	Rate of Change (Post-Intervention) DDD/1000-PD/month	Post minus Pre-Intervention Slope Change	P
Targeted Antibiotics	−0.92	−1.00	−0.08	0.821
Clindamycin	−0.19	0.04	0.23	<0.001
Ciprofloxacin	−0.03	0.44	−0.42	0.052
Moxifloxacin	−0.44	−0.44	−0.002	0.992
Metronidazole	−0.27	−0.19	0.08	0.536
Vancomycin	0.01	0.03	0.02	0.848
Control Antibiotics	−0.04	0.79	0.82	0.051
Amoxicillin-clavulanate	−0.15	0.30	0.45	0.061
Ceftriaxone	0.38	0.50	0.12	0.610
Piperacillin-tazobactam	−0.27	−0.01	0.26	0.140

**Table 3.** ITS Analysis (Level Change)

Antibiotic	Level (Pre-Intervention) DDD/1000-PD	Level (Post-Intervention) DDD/1000-PD	Post minus Pre-Intervention Level Change	P
Targeted Antibiotics	80.07	83.58	3.51	0.538
Clindamycin	5.10	5.62	0.52	0.482
Ciprofloxacin	27.34	26.39	−0.94	0.725
Moxifloxacin	11.96	16.98	5.02	0.013
Metronidazole	21.26	20.59	−0.66	0.482
Vancomycin	14.41	13.98	−0.42	0.817
Control Antibiotics	102.25	110.85	8.60	0.168
Amoxicillin-clavulanate	35.34	39.68	4.34	0.236
Ceftriaxone	42.50	41.14	−1.36	0.612
Piperacillin-tazobactam	24.41	30.03	5.62	0.013

## Discussion

Although the use of prescriber-entered indications to track antibiotic prescribing has been described in literature and has been shown to improve appropriate antibiotic prescribing,^4–7^ our institution is one of few hospitals in Canada that have adopted mandatory indications in practice. To our knowledge, this is the first study to evaluate the impact of prescriber-selected indications on antibiotic usage of targeted antibiotics and potential shifts in prescribing. The introduction of mandatory fields led to moderate uptake of the predefined indications. Additionally, the use of mandatory indications did not have any significant impact to overall targeted and control antibiotic prescribing. We hypothesized that in the context of an already established ASP and concurrent other ASP interventions such as electronic order sets on common infections and high-intensity prospective audit and feedback, the true impact of mandatory indications may have been diminished. At our institution, ciprofloxacin is listed as 3^rd^ line option for urinary tract infection and is not listed as an option for intra-abdominal infections. High-intensity prospective audit and feedback (PAF) at our institution comprise of twice weekly interdisciplinary rounds on our four internal medicine wards with a review of all internal medicine patients receiving any antimicrobial agent. High-intensity PAF was associated with a reduction in antibiotic use compared to our previous low-intensity PAF which consisted of ad-hoc review of patients on targeted antimicrobials.^10^ The antibiotic usage reduction from high-intensity PAF would have some overlap with the reduction in targeted antibiotics seen with mandatory indications. Additionally, perhaps more time was needed to see the impact of this intervention, given we saw more changes in trends than levels.

This study had several limitations. Given its retrospective design, unaccounted confounding factors may have mitigated the change in antibiotic usage. However, our time-series analysis accounted for seasonal and secular (consistent) trends in antibiotic use to reduce the impact of any confounder. Secondly, we did not evaluate the accuracy of indication selection nor appropriateness of therapy. However, previous studies have shown high accuracy of selected indication (74%–100%)^5–7,11^ for antimicrobials. We infer, based on these studies, that there was moderate to high accuracy and clinical appropriateness in the selection and use of our predefined mandatory indications. This finding was supported by the observations of specific infections noted when the free-text field was used and almost no incomprehensible rationale provided by prescribers.

Our next steps based on our findings include (1) optimizing the predefined list of indications to reflect the most commonly used free-form indications and (2) removing mandatory indications from oral vancomycin and indications pertaining to *Clostridioides difficile* infection from IV and oral metronidazole given the Infectious Diseases Society of America guideline update in 2017.
